# Early Impressions and Adoption of the AtriAmp for Managing Arrhythmias Following Congenital Heart Surgery

**DOI:** 10.21203/rs.3.rs-4125331/v1

**Published:** 2024-03-21

**Authors:** Scott M. Leopold, Diane H. Brown, Xiao Zhang, Xuan T. Nguyen, Awni M. Al-Subu, Krisjon R. Olson

**Affiliations:** American Family Children’s Hospital; Presbyterian Hospital; American Family Children’s Hospital; University of Wisconsin-Madison; American Family Children’s Hospital; American Family Children’s Hospital

**Keywords:** AtriAmp, PICU, arrhythmias, congenital heart surgery, diffusion of innovation theory, medical innovations

## Abstract

**Objective:**

AtriAmp is a new medical device that displays a continuous real-time atrial electrogram on telemetry using temporary atrial pacing leads. Our objective was to evaluate early adoption of this device into patient care, understand how it affected clinical workflow, and identify unforeseen benefits or limitations.

**Design:**

Qualitative study using inductive analysis of semi-structured interviews to identify dominant themes

**Setting:**

Single center, tertiary, academic 21-bed mixed pediatric intensive care unit (PICU)

**Subjects:**

PICU multidisciplinary team members (Pediatric intensivists, PICU Nurse Practitioners, PICU nurses and Pediatric Cardiologists) who were early adopters of the AtriAmp (n=14)

**Results:**

Three prominent themes emerged from qualitative analysis of the early adopters’ experiences. (1) Accelerated time from arrhythmia event to diagnosis, treatment, and determination of treatment effectiveness; (2) Increased confidence and security in the accuracy of providers’ arrhythmia diagnosis; and (3) Improvement in the ability to educate providers about post-operative arrythmias where reliance on time consuming consultation is a default. Providers also noted some learning curves with the device; none of which compromised medical care or clinical workflow.

**Conclusions:**

Insights from early adopters of AtriAmp signal the need for simplicity and fidelity in new technologies within the PICU. Further research in the qualitative and observational sphere is needed to understand how technologies, such as AtriAmp, find expanded use in the PICU environment. Our research suggests that such technologies can be pivotal to the support and growth of multi-disciplinary teams, even among those who do not participate in early implementation.

## Introduction

Advances in postoperative cardiac surgical care in the PICU have depended, in part, on medical technologies used to continuously monitor critically ill patients. Despite calls to do so, few studies focus on how and why technological advances are sustained within the PICU[1, 2]. With postoperative arrhythmias, no significant technological advances have occurred over the last few decades until recently[3–9]. AtriAmp (Atrility Medical, Madison, WI, USA) is a novel single-use device with continuous real-time and retrospective atrial electrogram monitoring capacity. This device has the potential to revolutionize the care of postoperative arrhythmias and decrease morbidity in these patients[10–16].

New technologies, however, can face significant barriers to adoption within the PICU. Diffusion of innovation theory suggests that adoption of new technologies follows a predictable pattern, with early adopters leading implementation[17, 18]. Thus, integral to the assessment of a new device like AtriAmp is understanding how early adopters perceive the device. This qualitative research aimed to evaluate adoption of AtriAmp into patient care, understand how it affected clinical workflow, and identify unforeseen benefits or limitations[19].

## Methods

We performed a qualitative study using grounded theory methods. We used an inductive analytical approach to evaluate how the AtriAmp affected patient care and workflows within a PICU with about 140 postoperative cardiac surgery admissions per year. Participants, whose interviews are the source data, were drawn from all levels of patient care including PICU nurses, PICU nurse practitioners (NP), pediatric intensivists, and pediatric cardiologists. A total sample of early adopters, defined as all of those on the multidisciplinary team who reported using the device within six months of its introduction, were interviewed and concepts that emerged during analysis were further developed to elaborate concepts under study. This iterative approach allows for the generation of explanatory models regarding adoption of the device grounded in empirical data about its use in pediatric intensive care. We continued to recruit participants and conduct interviews until no further early adopters could be identified.

One investigator (D.H.B), a pediatric critical care fellow, developed the interview guide and conducted audio-taped interviews in person between August and October 2021 lasting between 4–22 minutes; approximately 6 months after the device was first used on patients in our PICU and about 4–6 months before its use became the standard for postoperative cardiac care in our PICU. Interviews employed a semi-structured format (Supplemental Digital Content 1). The guide consisted of open-ended questions with targeted probes regarding: 1) experience and training in using the device, 2) how the device affected patient care, and 3) how the device affected clinical workflows. No repeat interviews were performed, and all participants spoke English. All interviews were transcribed verbatim.

Four members of the research team performed open, axial, and inductive coding of the interview transcripts. First, 3 authors (S.M.L., K.R.O, X.Z.) independently reviewed each transcript and open-coded for providers experiences and perspectives on early adoption (open-coding). The 3 authors met in person at regular intervals to review transcripts and codes, examine coding differences in coding and interpretation, and proximity to thematic saturation. This team included two pediatric critical care fellows (S.M.L. and D.B.) one medical anthropologist (K.R.O.), a graduate student in sociology (X.N.) a clinical research scientist in pediatric cardiology (X.Z.), and a pediatric intensivist (A.A.) whose backgrounds allowed diversity in interpretation of the data. We selected themes based on their prevalence and consistency in the data and a final schema describing early adoption practices (selective coding). We also performed member checking, in which study results were shared with study participants and their input was considered in the final analysis. NVivo 11 was used by K.R.O. to annotate transcripts, codes, and memos.

The University of Wisconsin-Madison institutional review board approval was obtained, and procedures were followed in accordance with the ethical standards of the institutional committee on human experimentation and with the Helsinki Declaration of 1975 (IRB2020–1146, approved on February 2, 2020, study title “Arrhythmia Identification using the AtriAmp”. Informed consent was obtained from all participants.

## Results

We approached 14 providers who self-identified as early adopters of Atri-Amp, all of whom provided informed consent, and participated in a semi-structured interview (Table 1).

Overall, the early adopters of the AtriAmp cited early adoption due to the device’s simplicity and fidelity, highlighting: 1) a reduction in time between arrhythmia event and diagnosis and/or treatment, 2) improved accuracy in arrhythmia diagnosis, 3) improved familiarity and education of atrial electrograms and postoperative arrhythmias and 4) some technical but easily overcome challenges. Exemplary quotations illustrative of these themes can be found in Table 2.

### Faster arrhythmia diagnosis and quicker time to treatment

**Figure 1:**
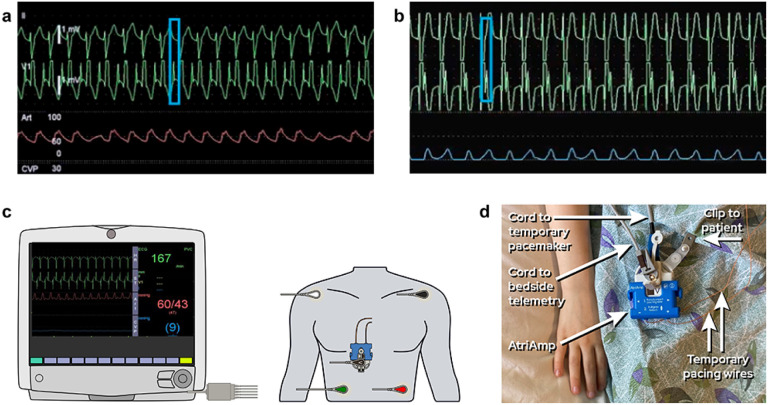
Demonstration of AtriAmp signals on telemetry and AtriAmp device hookup. (a) Sinus rhythm shown on the bedside telemetry with surface electrogram (top) and AtriAmp signal (bottom). (b) Junctional ectopic tachycardia shown on the bedside telemetry with surface electrogram (top) and AtriAmp signal (bottom). (c) Illustration of device hook up to patient and telemetry. {d) Image of device hooked up to actual patient

Participants described adopting the AtriAmp because it eliminated the cumbersome intermediate steps of acquiring an atrial electrogram. In doing so, it improved diagnostic efficiency while also avoiding episodes of hypotension that can occur due to delays in atrial electrogram acquisition. Once a treatment was started, the AtriAmp also allowed for better identification of treatment success or failure. Figure 1 demonstrates what the AtriAmp signal looks like in line with the surface electrogram on bedside telemetry and illustrates how the AtriAmp is connected to the patient and bedside monitor.

### More confident and accurate arrhythmia diagnosis

Early adopters we interviewed tended to view arrythmias within the broader context of their experience rather than being narrowly wedded to conventional biomarkers. They reported that through its continuous collection of atrial signals which are available for review both retrospectively and in real-time, the AtriAmp improves diagnostic accuracy. Those arrhythmia events that are episodic are still caught and can be reviewed, which may have been lost by the prior standard of care. Participants described that they were able to avoid diagnostic uncertainty, unnecessary treatments for wrong diagnoses, and iatrogenic treatments. Early adopters cited a preference for continuous rather than intermittent monitoring, and the AtriAmp is the first of its kind to provide higher fidelity continuous monitoring for postoperative arrhythmias. One pediatric intensivist went so far as to say that “it should become the new standard of care.” In line with this approach, study participants often said that Atri-Amp helped them to formulate more accurate treatment targets during a critical period. Benefits of the AtriAmp over previously used standards of care are shown in Table 3.

### Increased familiarity and improving education in atrial electrograms and post-op arrhythmias

Early adopters found the AtriAmp to have significant educational value. For less experienced providers, the device’s real time monitoring creates just-in-time learning opportunities around postoperative arrhythmias. In particular, the PICU NPs cited the device’s utility in creating these opportunities. A subset of these NPs noted a discernible learning curve regarding atrial electrogram interpretation, which led them to spend more time learning about all types of postoperative arrhythmias. Early adopters considered this especially important when the tendency of providers and the health system is to default to sub-specialty consultation, where technology can fill important gaps in knowledge and patient care.

### Technical challenges and learning curves

Participants noted several technical challenges involving device set-up, positioning and placement in the bed, controlling patient interactions, and confusion with pacing through the device. Importantly, providers noted that these pearning curves did not result in any perceivable difference in the quality of care provided to the patients and that all challenges were overcome with additional practice. These issues could be averted in the future through pre-use multidisciplinary educational sessions and/or training programs. Participants described acquiring skills in rhythm management and providing education to the team in order to educate those who did not adopt AtriAmp early. These findings highlight the need for more research to determine the acceptability, scalability, and outcomes of AtriAmp use in pediatric intensive care. Here we offer insights into the practice changes that support adoption of new technologies in the PICU.

## Discussion

While survival of critical illness, and particularly critical cardiac conditions, has improved dramatically we have limited data about how contributing technological innovations are disseminated and implemented in the PICU. In this study, we performed qualitative investigation into the initial impressions and issues encountered by early adopters of the AtriAmp, a new health technology device, in a pediatric intensive care unit on patients who are postoperative from cardiac surgery. In our study, the AtriAmp was overwhelmingly perceived as a significant improvement in patient care by all early adopters, regardless of their role in the PICU. When we spoke to this select group, common themes emerged around the device’s ease of use, accuracy, reliability, and educational utility.

The AtriAmp eliminated many of the cumbersome intermediate steps of acquiring an atrial electrogram following a postoperative arrhythmia event in the PICU. The providers interviewed in this study described that the new technology, by being continuously in line with the surface ECG, eliminated diagnostic difficulties with intermittent or episodic arrhythmias. In doing so, the AtriAmp accelerated the time between arrhythmia event and diagnosis, allowing for more rapid initiation of appropriate treatment and more rapid identification of whether the treatment had a beneficial effect. In addition, by using available resources more efficiently and focusing more time and energy on the patient rather than diagnosing an arrhythmia, many of pediatric intensivists described that AtriAmp created a safer and more secure environment for their patient. These findings are consistent with the literature on diffusion of innovation, which suggests that innovations that demonstrate a relative advantage, are compatible with the adopters’s need, and provide tangible results are most likely to diffuse through the population.

The AtriAmp also provided an unforeseen benefit as an educational tool for teaching about postoperative arrhythmias and in interpreting atrial ECGs. Many of the PICU NPs and PICU RNs commented on how their understanding of post-op arrhythmias and their ability to interpret atrial ECGs had improved since the introduction of the new device[19]. One of the PICU physicians also noted he was receiving more questions from the bedside RNs regarding things seen on the atrial waveform since initiation of the AtriAmp. Several providers commented on how ongoing education and review of atrial waveforms would benefit them. Learning and skill development are ongoing processes that are embedded in the PICU environment, and the use of the AtriAmp as an educational tool may have contributed to the development of new practices and habits in rhythm identification. These findings are consistent with the literature on early adopters of health technology, which suggests that these individuals often become opinion leaders and are more receptive to new technologies due to their intrinsic motivation to improve patient care and advance their own knowledge and skills[17].

Although all providers received a lecture on device setup and atrial ECG interpretation prior to the introduction of the AtriAmp, some unforeseen learning curves were encountered. The PICU RNs noted that it took some practice to get used to how to insert the temporary pacing wires into the device, and for neonates and infants, careful planning was required in positioning the device in the bed with the patient to avoid pulling on the wires or getting the patient tangled in the wires. However, all nurses were able to overcome these challenges with additional practice, and none of them felt like the device significantly interfered with their care of the patients. These findings are consistent with the literature on the adoption of new inventions in healthcare, which suggests that while new inventions may require some learning and adaptation, healthcare providers are generally receptive to new technologies that are relatively simple to learn how to use, can be easily experimented with, and demonstrate tangible improvements to patient care[20–22].

This study highlights the importance of understanding the environment in which new health technologies are introduced. Diffusion of innovation theory teaches that new healthcare technologies are most likely to be adopted if: 1) they demonstrate a relative advantage to the current standard of care, 2) they are compatible with the values and needs of the population, 3) they are relatively easy to understand and learn how to use, 4) they can be tested and experimented on and allow for adaptations, and 5) they produce tangible results[17, 18]. As demonstrated, the AtriAmp met all five of these criteria. While it is relatively unusual for new technologies to be readily adopted in the PICU, early adopters of different roles seized hold of various criterion and the learning healthcare environment created by AtriAmp. PICU RNs took more stock in the device’s simplicity and trialability, whereas the intensivists and cardiologists saw its relative advantage, compatibility with needs, and the results.

**Figure 2:**
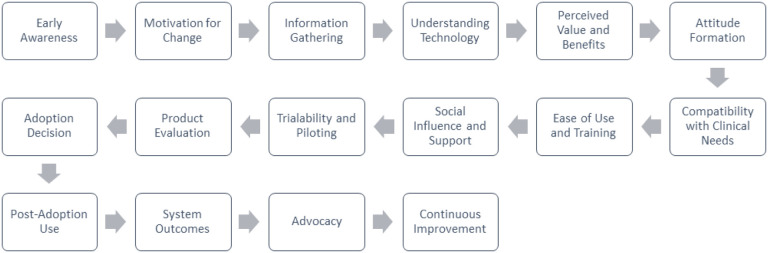
Steps in the adoption of new healthcare products and technologies within the hospital setting (adapted from diffusion of innovation theory)

Our analysis suggests that AtriAmp could expand to standard of care in pediatric critical care units like our own. However, for technological innovations to be widely adopted amongst all PICUs, there are additional factors that must be considered and additional hurdles that may be faced[23, 24]. The additional factors are outlined in Figure 2, an adaptation of diffusion of innovation theory, more specifically for the adoption of new healthcare products and technologies. The stages of awareness, information gathering, and understanding technology remain the same, but the subsequent stages have been modified to reflect unique considerations. For example, compatibility with clinical needs, ease of use and training, and social influence are all important factors in adopting medical technologies in the PICU.

Additionally, trialability and piloting have been added as a stage to reflect the importance of testing the technology in this setting before making an adoption decision. Finally, the diagram includes the importance of continuous improvement as a final stage, as ongoing evaluation and refinement of the technology are critical for long-term success in the PICU context.

The additional hurdles are outlined in Tables 4 and 5, which list several lessons that were learned from two previously adopted and now widely used healthcare technologies, the electronic health record (EHR) and near-infrared spectroscopy (NIRS).[25–29] Other barriers to widespread adoption of AtriAmp could include the lack of established learning health system infrastructure, ambivalence toward adoption outside of academic centers, or variability in staffing across PICUs.

This study has several limitations.[30–32] Because our goal was to learn about the experiences of early adopters who are at the forefront of a new technology introduced into the PICU, we purposively recruited a total sample of individuals with the most experience with AtriAmp. Consequently, this is not a representative sample and likely does not reflect the practices of all multidisciplinary PICU teams. In contrast, the early adopters studied here reflect the small size and gendered division of our PICU. Among our early adopters, the pediatric intensivists were all were male, while the NPs and RNs were all female. Previous research has shown that new technologies can reproduce gender inequities that impact women’s participation and change in health systems[33]. This may be underrepresented in our study, where the sample reflects the current gender imbalance of many multidisciplinary teams and especially in PICU leadership[34]. Second, our results reflect self-reported practices. Because we did not observe the providers using AtriAmp, we do not know to what extent self-reported practice reflects actual practice where robust observational research is an important future direction.

## Conclusions

The AtriAmp is an innovative tool that has the potential to significantly improve the management of arrhythmias following congenital cardiac surgery. The device provides valuable assistance in the management of postoperative arrhythmias, and it has the potential to significantly improve patient outcomes and enhance the quality of care in the pediatric ICU. Through qualitative research design, this study was able to identify how the AtriAmp fit into the PICU environment and why it is well set up for further dissemination in PICUs globally. Approximately 6 months after these interviews, the AtriAmp use became standard of care in these patients in our PICU. Since then, several additional PICUs and CICUs around the United States have begun experimenting with the device as well.

## Figures and Tables

**Table 1.** Study Participants’ Demographics (n =14)

Participant Characteristics	n = 14
Female, n	11
Clinical Profession, n total, n females	
PICU RN	5, 5
PICU NP	3, 3
Pediatric Intensivist	3, 0
Pediatric Cardiologist	3, 3
Years working in healthcare, mean (range)	
PICU RN	10 (6–15)
PICU NP	15 (13–19)
Pediatric Intensivist	28.3 (24–34)
Pediatric Cardiologist	25.7 (19–32)
Years working in an ICU, mean (range)	
PICU RN	10 (6–15)
PICU NP	14 (10–19)
Pediatric Intensivist	22 (20–24)
Number of Different Institutions worked at, mean (range)	
PICU RN	1.6 (1–4)
PICU NP	3 (2–4)
Pediatric Intensivist	4.33 (3–7)
Pediatric Cardiologist	3.33 (2–4)

**Table 2.** Notable Quotations from Interviews

Theme	Quotation
Reduced time to arrhythmia Diagnosis and Treatment	“I really think [it reduces] the time from arrhythmia to arrhythmia diagnosis… the time from diagnosis to the treatment… and the time to determine if you need to change treatment.” - Pediatric Cardiologist
	“[the AtriAmp] just takes a step out of the process of getting the ECG machine and getting a formal atrial electrogram” - Pediatric Cardiologist
	“it has really facilitated early recognition of atrial arrhythmias” -Pediatric Cardiac Intensivist
	“when we used to do atrial electrograms, by the time the EKG tech got up here, the patient may have been out of the rhythm and so we missed our opportunity to see what was going on” - PICU RN
More Accurate Arrhythmia Diagnosis	“use of the AtriAmp [has] allowed for more accurate diagnosis of postoperative arrhythmias.” - Pediatric Cardiac Intensivist
	“I always compare it to continuous pulse oximetry…It’s non-invasive and it gives you a ton of information.” - Pediatric Cardiologist
	“I think without the AtriAmp, patients would get treated for things that they didn’t need because we didn’t have an [AEG] available…. [I think] you would make assumptions and treat for the worst-case scenario” - PICU NP
	“it is by far the highest fidelity and most clear way to interpret the rhythm” - Pediatric Cardiac Intensivist
	“the AtriAmp gives you long term rhythm analysis rather than episodic” - Pediatric Cardiac Intensivist
	“I am definitely more confident in the heart rhythm when I have the AtriAmp in place” - Pediatric Cardiac Intensivist
	“the use of the AtriAmp should be the new standard of care” - Pediatric Cardiac Intensivist
Improves Familiarity and Education	“it’s allowed easier teaching of EP to residents, fellows, nurses…and it really has changed the ability for people to understand arrhythmias.” - Pediatric Cardiac Intensivist
	“it really makes the atrial signals so much more readable.” -Pediatric Cardiologist
	“it forces you to use atrial electrograms more.”- PICU intensivist
	“it’s really neat because it teaches people the value of having an [AEG].” - Pediatric Cardiac Intensivist
Technical Challenges and Learning Curves	“I just worry about pulling, but the clip (to attach to the patient) helps…we need to be more cognizant of how we are moving the patient”- PICU RN
	“I think it takes a little bit of time to figure out how to pull the clamp and lock the wires in”- PICU NP

**Table 3.** Comparison of the benefits of AtriAmp versus previous standards of care

	12 lead Atrial ECG	Bedside Surface ECG Monitoring	AtriAmp
Highest quality signal	**✓**		**✓**
Continuous and real-time		**✓**	**✓**
Accessible on telemetry		**✓**	**✓**
Historical review		**✓**	**✓**
Atrial pacing connection			**✓**

**Table 4.** Lessons from the adoption of the Electronic Health Record

Lesson	Comments
Usability	One of the biggest challenges with EHRs in the PICU has been usability. The complexity of the technology and the number of features it offers can be overwhelming, and clinicians may find it difficult to navigate the system efficiently. It is essential that AtriAmp is designed to be intuitive and user-friendly to avoid these issues.
Training and support	Clinicians need adequate training and ongoing support to use the EHRs effectively. The same applies to AtriAmp. Training sessions and continued support can ensure that users are confident in using the technology, which can lead to better adoption rates.
Interoperability	In the PICU, EHRs may not communicate with other systems, leading to problems with data transfer and patient care. AtriAmp should be designed to integrate seamlessly with existing EHRs and other clinical information systems to ensure that all data is accessible and accurate.
Data Security	Data security is a significant concern with EHRs in the PICU, and this is also relevant to AtriAmp. Robust security protocols must be put in place to protect patient data and ensure that only authorized users have access.
Change Management	The introduction of new technology can be disruptive, and change management is critical to ensure a smooth transition. It is essential to involve key stakeholders in the planning and implementation of AtriAmp to ensure buy-in and reduce resistance to change.

**Table 5.** Lessons from the adoption of Near Infrared Spectroscopy

Lesson	Comments
Address Concerns	Introducing new technologies can raise concerns among the users. In the case of NIRS, there were concerns regarding the accuracy of the readings, the comfort of the device, and the cost. These concerns were addressed through training and education, and by providing evidence of the benefits of using NIRS. Similar concerns may arise with the introduction of AtriAmp, and it is important to address them proactively.
Evaluate the impact	It is important to evaluate the impact of the new technology. In the case of NIRS, studies showed that it helped to improve patient outcomes, reduce the length of stay in the CICU, and reduce healthcare costs. The same approach could be taken when evaluating the impact of AtriAmp in the CICU.
Collaborate with Vendors	It is important to collaborate with vendors when introducing a new technology. Vendors can provide training and education, technical support, and assistance with implementation. In the case of NIRS, vendors worked closely with the hospital to ensure that the device was integrated into the workflow and that staff were trained on its use. The same approach could be taken when working with the vendor for AtriAmp.
